# ﻿Genetic identification, morphology and distribution of *Natrixhelvetica* subspecies in southern and western Switzerland (Reptilia, Squamata, Serpentes)

**DOI:** 10.3897/zookeys.1205.123911

**Published:** 2024-06-26

**Authors:** Andreas Schild, Hannes Baur, Stefan T. Hertwig, Uwe Fritz, Sylvain Ursenbacher

**Affiliations:** 1 Institute of Ecology and Evolution, University of Bern, Baltzerstrasse 6, 3012 Bern, Switzerland University of Bern Bern Switzerland; 2 Naturhistorisches Museum Bern, Bernastrasse 15, 3005 Bern, Switzerland Natural History Museum Bern Bern Switzerland; 3 Museum of Zoology (Museum für Tierkunde), Senckenberg Dresden, A. B. Meyer Building, 01109 Dresden, Germany Museum of Zoology (Museum für Tierkunde) Dresden Germany; 4 Department of Environmental Sciences, Section of Conservation Biology, University of Basel, Bernoullistrasse 32, 4056 Basel, Switzerland University of Basel Basel Switzerland; 5 info fauna – karch, University of Neuchâtel, Avenue de Bellevaux 51, 2000 Neuchâtel, Switzerland University of Neuchâtel Neuchâtel Switzerland; 6 Balaton Limnological Research Institute, 8237 Tihany, Klebelsberg Kuno u. 3, Hungary Balaton Limnological Research Institute Tihany Hungary

**Keywords:** Microsatellites, mitochondrial DNA, morphometrics, nuclear DNA, taxonomy

## Abstract

Most of Switzerland is inhabited by the nominotypical subspecies of the barred grass snake (*Natrixhelveticahelvetica*), which is characterized by mitochondrial DNA lineage E. Only in the northeast of the country, the common grass snake (*N.natrix*) occurs and hybridizes with *N.h.helvetica* in a narrow contact zone. However, we discovered that in southern and western Switzerland barred grass snakes representing another mtDNA lineage (lineage C) are widely distributed. Lineage C is typical for Alpine populations of the southern subspecies *N.h.sicula*. Our microsatellite analyses of the Swiss samples revealed differences between the two subspecies and also a substructure with two clusters in each subspecies. Furthermore, we discovered a contact and hybrid zone of *N.h.helvetica* and *N.h.sicula* along the northern shore of Lake Geneva and also confirm that interbreeding with alien common grass snakes (*N.n.moreotica*, mtDNA lineage 7) occurs there. This finding is of concern for nature conservation and measures should be taken to prevent further genetic pollution. Using morphometrics, we found no differences between the two subspecies of *N.helvetica*, while *N.natrix* was slightly distinct from *N.helvetica*.

## ﻿Introduction

Grass snakes constitute a complex of three species which were regarded as conspecific for many decades (Kabisch 1999; Pokrant et al. 2016; Kindler et al. 2017). *Natrixastreptophora* (Seoane, 1884) occurs in the North African Maghreb region, the Iberian Peninsula and adjacent France. *Natrixhelvetica* (Lacepède, 1789) lives in Western Europe north of the Pyrenees and in Britain and Italy. In Central Europe *N.helvetica* ranges eastward approximately to the Rhine region. *Natrixnatrix* (Linnaeus, 1758) occupies the largest distribution range of the three species, from east of the Rhine region across Fennoscandia, the Balkan Peninsula and large parts of the Near East to Lake Baikal in Central Asia. Traditionally, many grass snake subspecies have been recognized, based on often somewhat fuzzy morphological features such as body proportions, coloration and size (see reviews in Kabisch 1999; Kindler et al. 2013; Fritz and Schmidtler 2020). Most of these subspecies are currently no longer recognized (Kindler et al. 2018a; Schultze et al. 2020; Asztalos et al. 2021b). According to the current view (Asztalos et al. 2021b), two subspecies of *N.natrix* occur in Central Europe, the nominotypical subspecies *N.n.natrix* and *N.n.vulgaris* Laurenti, 1768. However, the two subspecies hybridize in large parts of southern and southeastern Central Europe, while the populations in northeastern Switzerland represent *N.n.vulgaris* with introgressed mitochondria of *N.n.natrix* (Asztalos et al. 2021a, 2021b). For *N.helvetica*, four subspecies are currently distinguished. *Natrixhelveticahelvetica* is distributed north of the Alps, while *N.h.sicula* (Cuvier, 1829) occurs south of the Alps, i.e., on the Italian Peninsula and on Sicily. Two further subspecies live in Corsica (*N.h.corsa* [Hecht, 1930]) and Sardinia (*N.h.cetti* Gené, 1839; Schultze et al. 2020). *Natrixhelveticasicula* crossed the Alps at least twice and occurs in the Inn River drainage of Austria and southernmost Bavaria (Glaw et al. 2019; Asztalos et al. 2021a). In addition, in Switzerland one record is known from beyond the Simplon Pass in the canton Valais (Kindler and Fritz 2018). *Natrixhelveticasicula* harbours several deeply divergent mtDNA lineages, reflecting ancient divergence processes on the Italian Peninsula and Sicily that began approximately 6.8 million years ago (Kindler et al. 2018a; Schultze et al. 2020). The populations of *N.h.sicula* relevant for the present study possess mtDNA lineage C (Schultze et al. 2020).

There are many regions across the distribution range of grass snakes where non-native individuals were introduced (France: Asztalos et al. 2020; Germany and Great Britain: Kindler et al. 2017; Italy: Schultze et al. 2020; Netherlands: van Riemsdijk et al. 2020; Asztalos et al. 2021c). Within the scope of a study in Switzerland, Dubey et al. (2017) genetically uncovered alien grass snakes (*N.natrix*) allegedly originating from Rijeka, western Croatia, which escaped in the 1970s from an outdoor reptile park in Lausanne (46.5427°, 6.6423°). However, the mtDNA lineage detected there (lineage 7 of Kindler et al. 2017, corresponding to *N.n.moreotica* [Bedriaga, 1882]) does not occur in the putative source region, but distinctly further southeast in the Balkans, western Anatolia and Cyprus (Asztalos et al. 2021b). Additionally, Dubey et al. (2017) found grass snakes yielding mtDNA lineage C at the same site in Lausanne. In contrast, Chèvre (2015) and Kindler and Fritz (2018) detected this lineage only in the cantons of Valais and Ticino, raising the question of whether these records also refer to non-native snakes or whether clade C, i.e., *N.h.sicula*, might have a wider distribution in Switzerland.

Dubey et al. (2017) assumed that grass snakes with lineage C also escaped from the reptile park and established in the region. However, it is also possible that *N.h.sicula* had passed the Alps in the Holocene and naturally occurs in western Switzerland. As grass snakes of clades C (*N.h.sicula*) and 7 (*N.n.moreotica*) have potentially hybridized at Lausanne with the native *N.h.helvetica* (clade E), it is of utmost importance for nature conservation to find out whether clade C is also native in western Switzerland or has been introduced.

The aim of the present study is to determine the natural distribution of *N.h.sicula* in Switzerland. To do so, we collected DNA samples from wild snakes and museum specimens to determine their mtDNA lineage. In addition, we used nuclear DNA markers (microsatellites) for Bayesian cluster analyses to estimate the amount of admixture between snakes corresponding to different mitochondrial lineages. In accordance with the concept of integrative taxonomy, we also used various characters of pholidosis and colour pattern as well as morphometric measurements to identify possible external morphological differences in our sample.

## ﻿Materials and methods

### ﻿Study area and sampling

**Figure 1.**
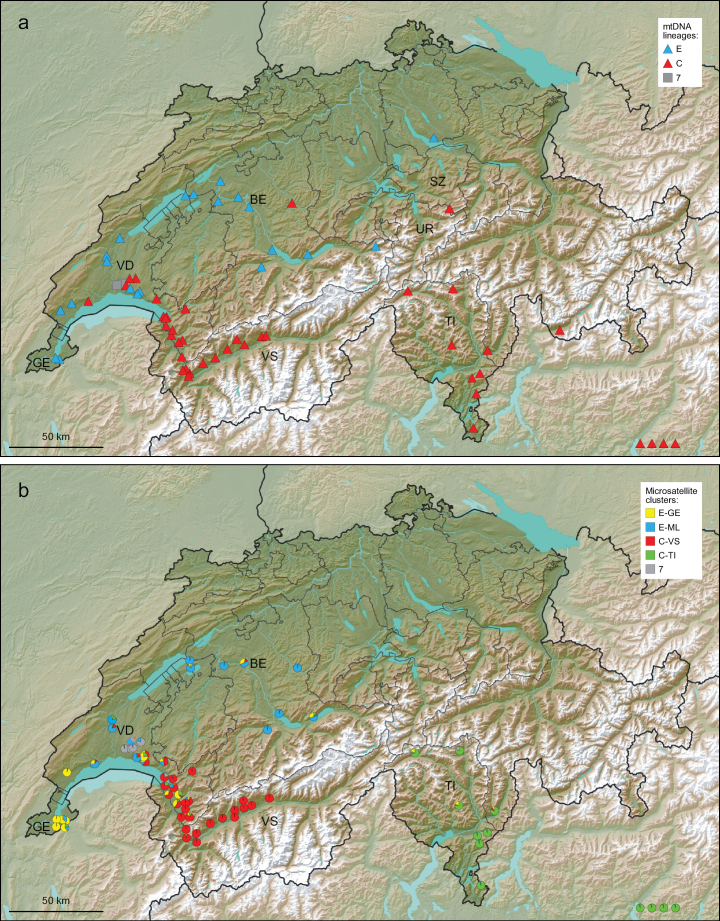
Distribution of mitochondrial lineages of grass snakes in southern and western Switzerland (**a**) and microsatellite clusters according to our STRUCTURE analyses (**b**). Symbols and colours of mitochondrial lineages correspond to Kindler et al. (2013). Some symbols are slightly shifted from the sampling location, enhancing readability. The four samples in the bottom right corner originate from northern Italy beyond the map sector. Borders within Switzerland denote cantons. Abbreviations for cantons mentioned in the text: BE – Bern, GE – Geneva, SZ – Schwyz, TI – Ticino, UR – Uri, VD – Vaud, VS – Valais. Lake Geneva, mentioned in the text, is located in the southwesternmost part of Switzerland (cantons of Geneva and Vaud) and adjacent France. The maps were created by modifying a Wikimedia map (https://commons.wikimedia.org/wiki/File:Reliefkarte_Schweiz2.png).

The immediate study area was limited to southern and western Switzerland. It comprised the cantons of Vaud, Valais and Ticino (Fig. 1) as lineage C has been detected there before (database of info fauna – karch; https://www.infofauna.ch/). The sampling scheme was habitat-specific, i.e., we focused on preferred habitats of grass snakes, like ponds, rivers, lakes and spots with previous sightings. Snakes were caught by hand, measured on site, and released directly afterwards at the exact location of capture. DNA samples were taken with buccal swabs (dried and stored at -20 °C) or scale clipping (one to four ventral scales stored in 70% alcohol). Following Thorpe (1979) and Chèvre (2015), individuals with a total length of less than 50 cm were classified as juveniles. To reduce handling time, all necessary body parts of adult snakes were photographed for later morphological analyses. Furthermore, museum specimens from Vaud, Valais and Ticino were studied; a few additional individuals from the adjacent cantons were also included. From museum specimens, liver or muscle tissue was sampled. A list of samples is provided in Suppl. material 1: table S1.

### ﻿DNA extraction and purification

Samples were incubated with ATL buffer and proteinase K (Qiagen) in a heat block for 16–20 h at 56 °C. Scales were previously placed in water for 24 h to remove alcohol. After digestion, liquid from swab tips was extracted using a centrifuge. The DNA was purified following the protocol “Purification of total DNA from Animal Tissues (Spin-Column Protocol)” of the DNeasy Blood and Tissue Kit (Qiagen) using a Qiagen robot.

### ﻿mtDNA sequencing

To determine the mtDNA lineage, the cytochrome *b* gene (cyt *b*) and the NADH dehydrogenase subunit 4 gene with adjacent regions coding for tRNAs (ND4) were used, as in previous studies on grass snakes (e.g., Kindler et al. 2013, 2017; Chèvre 2015; Pokrant et al. 2016; Glaw et al. 2019; Schultze et al. 2020; Asztalos et al. 2021a, 2021b). Amplification followed Kindler et al. (2013) and Chèvre (2015). PCR products were sequenced by LGC Genomics GmbH (Berlin, Germany). Sequences were processed with CodonCode Aligner (https://www.codoncode.com) and compared with GenBank sequences to determine the mitochondrial lineage.

### ﻿Microsatellite analyses

The same thirteen microsatellite loci were used as in Chèvre (2015) and Kindler et al. (2017) and genotyped according to their protocols. PCR products were analysed on an ABI 3130xl Genetic Analyser (Applied Biosystems) at the Zoological Institute of the University of Basel. The Microsatellite Plugin in GENEIOUS PRIME 2020.0.3 (https://www.geneious.com) was used to visualize peaks and determine allele lengths.

For inferring the nuclear genomic identity of the 73 successfully processed samples, the Bayesian clustering approach based on the Monte Carlo Markov chain (MCMC) algorithm implemented in the software STRUCTURE ver. 2.3.4 (Pritchard et al. 2000; Pritchard and Wen 2002) was used. STRUCTURE assumes unlinked microsatellite loci at linkage equilibrium and divides the dataset into partitions (*K*) optimized for the presence of Hardy-Weinberg equilibrium. After a burn-in of 100,000 generations, MCMCs were run for 200,000 iterations, ten times per *K* between one and ten. The optimal number of *K* was determined using both the Δ*K* method (Evanno et al. 2005) in STRUCTURE HARVESTER software (Earl and vonHoldt 2012) and the L(*K*) approach of Pritchard and Wen (2002). The best STRUCTURE run (highest likelihood) with the optimal *K* was used both to determine genotypic identity and to assess admixture. Snakes with an assignment ≥ 80% to a specific cluster were treated as pure. *F*_ST_ values between clusters were calculated with FSTAT ver. 2.9.3 (Goudet 1995) using only genotypically non-admixed individuals.

### ﻿Morphological analyses

Only snakes exceeding 50 cm in total length and with a genotypic cluster assignment ≥ 80% were used for morphological examinations. Following Chèvre (2015), morphological variables with strong geographic variation were selected (Suppl. material 1: table S2) and analysed along with landmark data to examine for possible morphological differences between microsatellite clusters. The dataset was enriched with additional data from photographs provided by M. Chèvre (21 genotyped *N.h.helvetica*, mtDNA lineage E; 20 genotyped *N.n.vulgaris*, mtDNA lineage 3 from northeastern Switzerland), so that 31 males, 38 females and three sex-undetermined grass snakes were available for morphology.

### ﻿Geometric morphometrics of landmark data

To obtain landmark data, standardized pictures were used showing the right and dorsal sides of the head of adult snakes. Fixing the focal length and manual focus of the camera ensured that the scale of the pictures was identical, which was double-checked using a ruler in the pictures. Photographs were taken twice to calculate mean landmark coordinates, which reduces potential inaccuracies due to slight shifts in photographing and placing landmarks. M. Chèvre provided only a single photograph per snake, for which landmark coordinates were produced twice to account for imprecise landmark placing. Mostly easily identifiable junctions of scales were chosen as landmarks to facilitate the workflow (Suppl. material 1: fig. S1).

Some landmarks were removed for analysis because the sample size was too small for 27×2 coordinate variables. Landmark 8 was removed because the temporal scale was sometimes divided and/or small, so it did not reach the 7^th^ supralabial scale. Landmarks 12, 14, 16 and 18 were excluded as they all have other landmarks in close proximity. Lastly, landmarks 21, 24 and 27 were removed because they are located at the edge and might already be influenced by the curvature of the head. Therefore, only nineteen landmarks (1–7, 9–11, 13, 15, 17, 19, 20, 22, 23, 25, 26) were finally used (Suppl. material 1: figs S3, S4).

Landmarks were placed in the software TPSDIG2 ver. 2.30 (Rohlf 2017) and its coordinates were saved in tps files created by the software TPSUTIL32 ver. 1.74 (Rohlf 2013). In the statistical software R ver. 3.4.1 (R Core Team 2017), the function *estimate.missing* () from the package GEOMORPH (Adams et al. 2019) was used to interpolate missing landmarks with the thin-plate spline method. Then, mean values were calculated for each landmark per specimen and side.

All analyses of the mean landmark coordinates were performed in MORPHOJ ver. 1.07a (Klingenberg 2011), similar to the procedure described in Sidlauskas et al. (2011). First, a least-square Procrustes Fit was calculated. Allometric correction was then performed using a linear regression with the log centroid size as explanatory variable and the Procrustes coordinates as the response variable. The regression included a permutation test with 10,000 rounds and pooled regression within clusters. To examine shape changes, the regression residuals were included in a Canonical Variate Analysis (CVA) with a permutation test for pairwise distances of 10,000 iterations. Scatterplots of CV scores were checked for clustering of groups and wireframe graphs were used to visualize shape changes. The starting and target shape of wireframe graphs were placed next to each other, as suggested by Klingenberg (2013), to be able to objectively examine shape changes. Besides visually plotting the shape differences, the CVA also runs both Mahalanobis and Procrustes permutation tests (10,000 iterations) to check for the significance of shape differences.

### ﻿Analysis of distance measurements

Distance measurements (SVL, TL, HL and HW; Suppl. material 1: table S2) were taken in the field and analysed using Multivariate Ratio Analysis (Baur and Leuenberger 2011; Baur 2024). The ‘shape PCA’ enables the examination of shape changes depending on size. The ‘PCA ratio spectrum’ then allows the interpretation of principal components (PCs) in terms of ratios and shows the most discriminating ratio with respect to a particular shape PC. This approach has been used in several studies to find morphological differences among taxa (László et al. 2013; Baur et al. 2014; Huber and Baur 2016; Gebiola et al. 2017; Waser et al. 2017). The package MICE (van Buuren and Groothuis-Oudshoorn 2011) was used to replace missing values through multiple imputation within variable groups after excluding individuals with > 25% missing data.

### ﻿Analysis of scale counts and colour markings

Scale counts (VS, SCS, PTS and GS; Suppl. material 1: table S2) taken in the field or from photographs were analysed using a standard PCA on the correlation matrix of the data. Colour markings (LBS, LBL, LBW, NMS, NMW, NMUC, NMLC and NMPS; Suppl. material 1: table S2) were quantified with the number of coloured scales as the unit and analysed using a standard PCA on the correlation matrix of the data.

### ﻿Linear discriminant analysis

Distance measurements, scale counts, measures for colour markings and three additional variables (RelRedPos, TPOS and BW; Suppl. material 1: table S2) were compared in a linear discriminant analysis to check whether microsatellite clusters are distinguishable. The function *lda* () from the package MASS (Venables and Ripley 2002) was used with equal priors and CV=TRUE. This was repeated for samples of *N.helvetica* only to test whether the discrimination could be improved.

## ﻿Results

### ﻿Distribution of mtDNA lineages

The distribution of mtDNA lineages in southern Switzerland is shown in Fig. 1a (for details of each sample, see Suppl. material 1: table S1). The cantons of Ticino and Valais are solely inhabited by *Natrixhelvetica* with lineage C, typical for Alpine *N.h.sicula*. In contrast, the snakes in the cantons north of the Alps harbour mainly lineage E, typical for *N.h.helvetica*. However, north of the Alps a few lineage C individuals were recorded as well (cantons of Bern, Schwyz and Vaud). The northern shore of Lake Geneva seems to be a broad contact zone of lineages C and E. In addition, in this region, in Lausanne, some *N.n.moreotica* (mtDNA lineage 7) were caught, as already described by Dubey et al. (2017).

### ﻿Microsatellite clusters

According to Pritchard and Wen (2002), the optimal number of clusters *K* has the highest likelihood L(*K*) value, which is here *K*=5 (Suppl. material 1: fig. S2, top left). In contrast, the Δ*K* method (Evanno et al. 2005) revealed *K*=2 as the best solution but also inferred a second pronounced peak for *K*=5 (Suppl. material 1: fig. S2, bottom right). The Δ*K* method reliably identifies the uppermost hierarchical level of genetic partitioning (Evanno et al. 2005); in our case, this corresponds to samples representing the two species of grass snake, *N.helvetica* and *N.natrix*. For inferring genotypic partitions (clusters) within *N.helvetica* and *N.natrix*, either subsets corresponding to each species can be examined separately in STRUCTURE or the STRUCTURE runs using the highest L(*K*) value can be inspected. Considering the highest L(*K*) value and the second peak of the Δ*K* approach, we present here the results for STRUCTURE runs using *K*=5. These five clusters correspond in our dataset to one cluster for *N.natrix* (i.e., genotypes of *N.n.moreotica* from Lausanne plus *N.n.vulgaris* from northeastern Switzerland) and four clusters within *N.helvetica* (for details, see Suppl. material 1: table S1).

Fig. 1b shows the geographic distribution of the five microsatellite clusters for western and southern Switzerland. One cluster (grey in Fig. 1b) represents the alien *N.n.moreotica* with mtDNA lineage 7 from Lausanne. *Natrixhelveticasicula* (mtDNA lineage C) is divided into two clusters, one in Valais (C-VS, red in Fig. 1b) and another one in Ticino (C-TI, green in Fig. 1b). Translated into the hydrographic net, the green cluster is found in river valleys connected to the great pre-Alpine Italian lakes and the Po drainage, and the red cluster is confined to the eastern (i.e., Alpine) Rhone drainage, from eastern Lake Geneva upstream. *Natrixhelveticahelvetica* (mtDNA lineage E) is also divided into two clusters, one in the Swiss Plateau (Mittelland; E-ML, blue in Fig. 1b) and another cluster along Lake Geneva (E-GE, yellow in Fig. 1b). Admixture between clusters is evident in particular, but not only, in geographic contact zones, mainly the Lake Geneva region and the adjacent Rhone valley near Montreux. Most of these admixed snakes are *N.helvetica* (admixture among the four respective microsatellite clusters; 16 individuals from the cantons Geneva, Bern, Vaud and Ticino; Suppl. material 1: table S1). Another *N.helvetica* from the canton Bern (Langnau im Emmental) shows mito-nuclear discordance. This snake harbours a mitochondrial haplotype of lineage C combined with a microsatellite assignment to cluster E-ML. However, there are two further snakes with mito-nuclear discordance resulting from hybridization of *N.helvetica* with the alien *N.n.moreotica* in the region of Lausanne. These two snakes are having predominantly *N.natrix* genotypes combined with mitochondrial haplotypes of *N.helvetica* (lineage C; Suppl. material 1: table S1).

Genetic differentiation (*F*_ST_) values are similar between clusters, except for the slightly lower values for E-GE/E-ML and for E-ML/*natrix*, whereas the highest value was observed between C-VS/E-GE (Suppl. material 1: table S3).

### ﻿Morphology

For morphological and landmark analyses, snakes representing the clusters E-ML and E-GE were merged in one cluster E, while C-VS and C-TI were kept separate. C-VS and C-TI are geographically divided by mountainous regions difficult to cross for grass snakes, whereas E-ML and E-GE are in contact and admixing. Additionally, the number of samples for E-GE is very limited.

**Figure 2.**
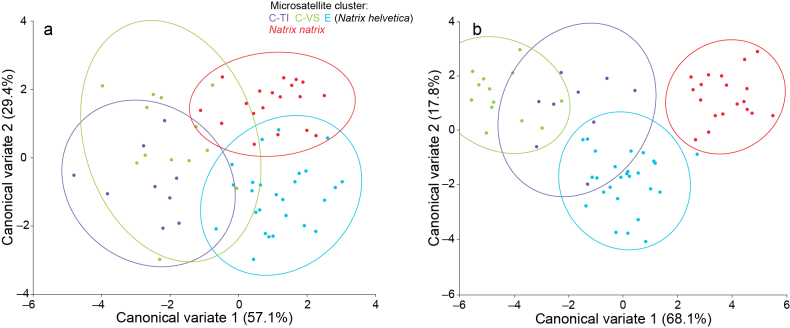
Canonical Variate Analysis (CVA) of dorsal (**a**) and lateral (**b**) landmark coordinates for grass snakes assigned to the microsatellite clusters of *Natrixhelvetica* (E, C-VS, C-TI) and *N.natrix* (*N.n.vulgaris*, mtDNA lineage 3, from northeastern Switzerland). Only individuals with a genotypic cluster assignment ≥ 80% are included; circles represent 95% confidence ellipses.

Mahalanobis and Procrustes permutation tests revealed significant morphological differences between all clusters (Table 1). However, Fig. 2 shows that there is always some overlap among all clusters, except for the lateral landmarks, where *N.natrix* was distinct. Also, there are no obvious shape differences visible in the wireframe graphs (Suppl. material 1: figs S3, S4).

**Table 1.** Statistical test results of Canonical Variate Analysis (CVA) of lateral and dorsal landmarks (Fig. 2) using 10,000 permutations for each test. E, C-VS and C-TI represent *Natrixhelvetica* clusters derived from microsatellite analyses (Fig. 1); *natrix* refers to samples from northeastern Switzerland (*N.n.vulgaris*, mtDNA lineage 3).

**Lateral landmarks**							
**Mahalanobis distances among groups**				**Procrustes distances among groups**			
	*natrix*	E	C-VS		*natrix*	E	C-VS
E	4.8305			E	0.0308		
C-VS	8.2776	5.6166		C-VS	0.0380	0.0285	
C-TI	6.5247	4.5882	5.2595	C-TI	0.0431	0.0324	0.0237
**P values from permutation tests**				**P values from permutation tests**			
	*natrix*	E	C-VS		*natrix*	E	C-VS
E	< 0.001			E	< 0.001		
C-VS	< 0.001	< 0.001		C-VS	< 0.001	< 0.001	
C-TI	< 0.001	< 0.001	< 0.001	C-TI	< 0.001	< 0.001	0.0783
**Dorsal landmarks**							
**Mahalanobis distances among groups**				**Procrustes distances among groups**			
	*natrix*	E	C-VS		*natrix*	E	C-VS
E	2.7561			E	0.0232		
C-VS	3.5488	3.7053		C-VS	0.0236	0.0268	
C-TI	4.4217	4.1624	2.9620	C-TI	0.0375	0.0389	0.0264
**P values from permutation tests**				**P values from permutation tests**			
	*natrix*	E	C-VS		*natrix*	E	C-VS
E	< 0.001			E	< 0.001		
C-VS	< 0.001	< 0.001		C-VS	< 0.001	< 0.001	
C-TI	< 0.001	< 0.001	< 0.001	C-TI	< 0.001	< 0.05	< 0.001

**Figure 3.**
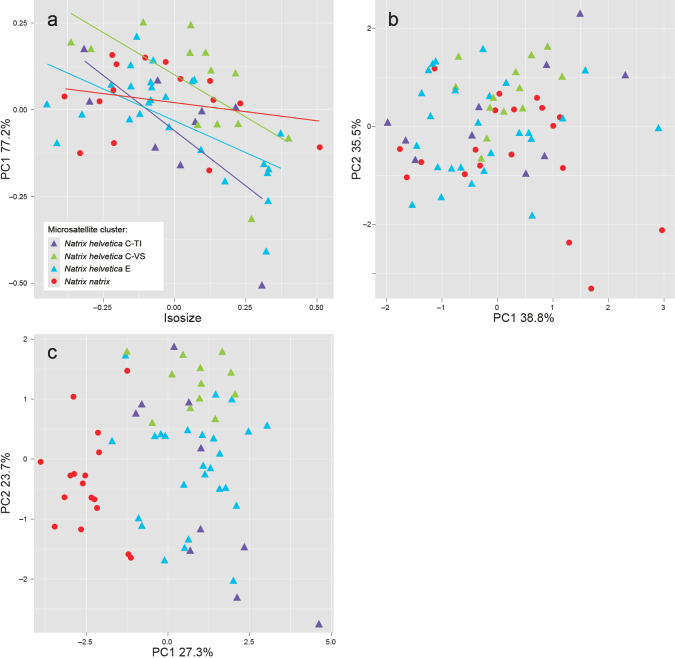
Shape PCA of distance measurements (**a**), standard PCA of scale counts (**b**) and standard PCA of colour marking measurements (**c**) for grass snakes assigned to the microsatellite clusters of *Natrixhelvetica* (C-TI, C-VS, E) and *N.natrix* (*N.n.vulgaris*, mtDNA lineage 3, from northeastern Switzerland). Only individuals with a genotypic cluster assignment ≥ 80% are included.

Shape PCA and standard PCAs show no differentiation of mtDNA lineages E, C-VS and C-TI of *N.helvetica* (Fig. 3). Only *N.natrix* is slightly distinct because of smaller and differently shaped markings. Similar results are obtained for the linear discriminant analysis. *Natrixnatrix* also represents here the most distinct cluster with a percentage of 86.7% of correctly allocated individuals. For all other clusters, the percentages are below 70% (Suppl. material 1: table S4). Mean values and standard deviations of each morphological trait are summarized for the two *N.helvetica* subspecies in Suppl. material 1: table S5.

## ﻿Discussion

After the discovery of two putatively alien mtDNA lineages of grass snake around Lausanne (lineages C and 7 of Kindler et al. 2013), concerns were raised about genetic pollution of native populations of *Natrixhelvetica* (Dubey et al. 2017). It was clear that the common grass snakes harbouring mtDNA lineage 7, now identified with the subspecies *N.natrixmoreotica* (see Asztalos et al. 2021b), are allochthonous and originate from the former Vivarium at Lausanne. However, for the other mitochondrial lineage (C), typical for Alpine representatives of *N.helveticasicula* (Schultze et al. 2020; Asztalos et al. 2021a), there remained the possibility of a natural occurrence. Dubey et al. (2017) also assumed that grass snakes with clade C had escaped from the Vivarium in the 1970s, together with the ancestors of what is now called *N.n.moreotica* (in Dubey et al. 2017, the inappropriate name *N.n.persa* is still used; see Asztalos et al. 2021b). However, the Alpine lineage of *N.n.sicula* is widely distributed in northern Italy, including the Alps, and its range extends northwards across the Alps to Tyrol and southernmost Bavaria (Glaw et al. 2019; Schultze et al. 2020; Asztalos et al. 2021a; Neumann et al. 2024). Until recently, only two records for *N.n.sicula* from Switzerland were published, one from Ticino and the other from beyond the Simplon Pass (Niedergesteln, Valais; Kindler and Fritz 2018; Schultze et al. 2020). Yet, these records and the wide Alpine distribution of *N.h.sicula* suggest that this subspecies could not only occur naturally in Switzerland, but that it has a much wider distribution than previously thought. This is supported by our present investigation.

Our study shows that *N.h.sicula* is widely distributed in the cantons of Ticino and Valais and ranges to the canton of Vaud, along Lake Geneva, where it hybridizes with *N.h.helvetica*, as evidenced by microsatellite genotypes. Therefore, *N.h.sicula* should no longer be considered as non-native around Lausanne, as supposed by Dubey et al. (2017) for the records of mtDNA lineage C there.

The two microsatellite clusters of *N.h.helvetica* and *N.h.sicula* correspond to local population structure. It is possible that our cluster E-GE (Fig. 1b) matches the southern cluster revealed by Asztalos et al. (2020), who also identified two distinct clusters for *N.h.helvetica*. Their southern cluster from France could reach upstream in the Rhone Valley to Lake Geneva, whereas our cluster E-ML could match the northern cluster of Asztalos et al. (2020). However, the sample from Geneva in Asztalos et al. (2020) was also assigned to the northern group and the differentiation of the cluster E-GE could instead also be related to a local effect (isolation by distance) and the tendency of STRUCTURE to cluster samples with similar ancestry. In contrast, until now, no substructure has been described for Alpine *N.h.sicula*, but the distribution pattern of our two clusters C-VS and C-TI makes sense biogeographically. Each cluster matches another drainage system (Alpine Rhone Valley vs. Po drainage).

North of Lake Geneva, we were not only able to detect the natural contact and hybridization zone of the two subspecies of *N.helvetica*. Our microsatellite data (Fig. 1b; Suppl. material 1: table S1) also confirm genetic pollution of *N.helvetica* from alien *N.n.moreotica*. Nature conservation should take action to prevent wider introgression of alien genes in this region. Non-native snakes and their hybrids should be removed before they further compromise the genetic identity of the native populations of *N.helvetica*, which is currently classified as “endangered” in the most recent Swiss Red List of Reptiles (BAFU & info fauna 2023).

The distribution and hybrid zone of *N.h.helvetica* and *N.h.sicula* in Switzerland can be explained in a biogeographical context. Kindler et al. (2018b) suggested that the nominotypical subspecies survived the last glaciation in southern France, from where it expanded its range to more northern regions during the Holocene warming. *Natrixhelveticasicula*, on the other hand, was inferred to have survived the last glaciation in a distinct ‘microrefugium’ in northeastern Italy (Kindler et al. 2013; Schultze et al. 2020), but it was impossible to endure in the Alpine Rhone Valley, which was completely covered by ice during the Late Glacial Maximum (Seguinot et al. 2018). As a consequence of the Holocene warming, *N.h.sicula* crossed the main Alpine chain not only in the east to Tyrol and southern Bavaria (Glaw et al. 2019; Asztalos et al. 2021a), but also in the west, as we know now. There, it established a secondary contact and hybrid zone with *N.h.helvetica* along Lake Geneva. This scenario implies transalpine dispersal at altitudes of about 2000–2200 m a.s.l., i.e., at altitudes that were only very recently colonized by the species (database of info fauna – karch, which hosts more than 25,000 records for grass snakes in Switzerland and is the national reference centre for Swiss amphibians and reptiles). Thus, it can be speculated that *N.h.sicula* crossed the western Alps during a Holocene period, which was at least as warm as present, probably via the Simplon Pass (approx. 2000 m a.s.l.).

In this context, two other northern records of grass snakes with lineage C are difficult to interpret (Langnau im Emmental, canton Bern, and Muotathal, canton Schwyz, directly at the border to canton Uri). For the snake from Muotathal no genotype is available, but the snake from Langnau im Emmental is, according to its microsatellite genotype, a pure representative of the cluster E-ML. Also, two other snakes from the canton Bern have an admixed genotype (Fig. 1; Suppl. material 1: table S1), suggesting that some *N.h.sicula* left indeed their genetic footprint there. It is well known that grass snakes are spread with building material, etc. or have been voluntarily moved (e.g., Ahnelt et al. 2021; Asztalos et al. 2021c), so these northern records are not necessarily evidence for wide-reaching natural dispersal and introgression across the Alps.

In contrast to genetic data, our morphological analyses revealed only a weak differentiation among the studied grass snakes. Only the two species *N.helvetica* and *N.natrix* could be morphologically discriminated with some confidence, while the used traits were not helpful in discriminating the two subspecies of individual genetic clusters within *N.helvetica*. This does not contradict the validity of the involved two subspecies because morphological traits that can be distinguished by humans are neither necessarily biologically relevant nor a prerequisite for taxonomic distinctness (compare, for instance, Kindler and Fritz 2018; Dufresnes et al. 2023, 2024). Also, we could have missed some relevant traits in coloration and pattern that were only recently highlighted (Fritz et al. 2023). According to these authors, some individuals of *N.h.sicula* show a “spotted” colour pattern that never occurs in the nominotypical subspecies. It cannot be excluded that further traits exist that help to identify the different subspecies even in the field, but it remains a challenge to disentangle this situation.

## ﻿Additional information

### Author ORCIDs

Hannes Baur https://orcid.org/0000-0003-1360-3487

Stefan T. Hertwig https://orcid.org/0000-0003-1617-8180

Uwe Fritz https://orcid.org/0000-0002-6740-7214

Sylvain Ursenbacher https://orcid.org/0000-0001-5093-6403

### Data availability

All data that support the findings of this study are available in the main text or the Suppl. material. No new haplotypes were identified in the present study, which is why no sequences were uploaded to the European Nucleotide Archive (ENA).

## Supplementary materials

Supplementary material 1 Supplementary information Data type docx Explanation note **fig. S1.** Landmark positions. **fig. S2.** STRUCTURE HARVESTER results indicating the optimal number of microsatellite clusters (K). **fig. S3.** Wireframe graph showing shape changes along the canonical variate 1 (CV1) for dorsal landmarks. **fig. S4.** Wireframe graph showing the shape changes along the canonical variate 1 (CV1) for lateral landmarks. **table S2.** Morphological variables measured. Illustrations from Chèvre (2015). **table S3.** Pairwise *FST* values between microsatellite clusters of *Natrixhelvetica* (E-GE, E-ML, C-VS and C-TI) and *N.natrix*. **table S4.** Morphological assignment of specimens (horizontal rows) to microsatellite clusters of *Natrixnatrix* and *N.helvetica* (E, C-VS, C-TI, vertical columns) based on all morphological variables using a Linear Discriminant Analysis (LDA). **table S5.** Mean and standard deviation (SD) of different morphological traits for Swiss barred grass snakes (*Natrixhelvetica*). https://binary.pensoft.net/file/1079849This dataset is made available under the Open Database License (http://opendatacommons.org/licenses/odbl/1.0/). The Open Database License (ODbL) is a license agreement intended to allow users to freely share, modify, and use this Dataset while maintaining this same freedom for others, provided that the original source and author(s) are credited.Andreas Schild, Hannes Baur, Stefan T. Hertwig, Uwe Fritz, Sylvain Ursenbacher
