# Supplementary material for: ﻿Genetic identification, morphology and distribution of Natrixhelvetica subspecies in southern and western Switzerland (Reptilia, Squamata, Serpentes)
Source: Zookeys. 2024 Jun 26;1205:223–38. doi: 10.3897/zookeys.1205.123911 (PMC11222720; doi:10.3897/zookeys.1205.123911)
Supplement: Supplementary material 1 — Supplementary information [file zookeys-1205-223_article-123911__-s001.docx]

**Genetic identification, morphology and distribution of *Natrix helvetica* subspecies in southern and western Switzerland**

Andreas Schild^1^, Hannes Baur^1,2^, Stefan T. Hertwig^1,2^, Uwe Fritz^3^, Sylvain Ursenbacher^4,5,6^

1 *Institute of Ecology and Evolution, University of Bern, Baltzerstrasse 6, 3012 Bern, Switzerland*

2 *Naturhistorisches Museum Bern, Bernastrasse 15, 3005 Bern, Switzerland*

3 *Museum of Zoology (Museum für Tierkunde), Senckenberg Dresden, A. B. Meyer Building, 01109 Dresden, Germany*

4 *Department of Environmental Sciences, Section of Conservation Biology, University of Basel, Bernoullistrasse 32, 4056 Basel, Switzerland*

5 *info fauna – karch, University of Neuchâtel, Avenue de Bellevaux 51, 2000 Neuchâtel, Switzerland*

6 *Balaton Limnological Research Institute, 8237 Tihany, Klebelsberg Kuno u. 3, Hungary*

**Supplementary material**


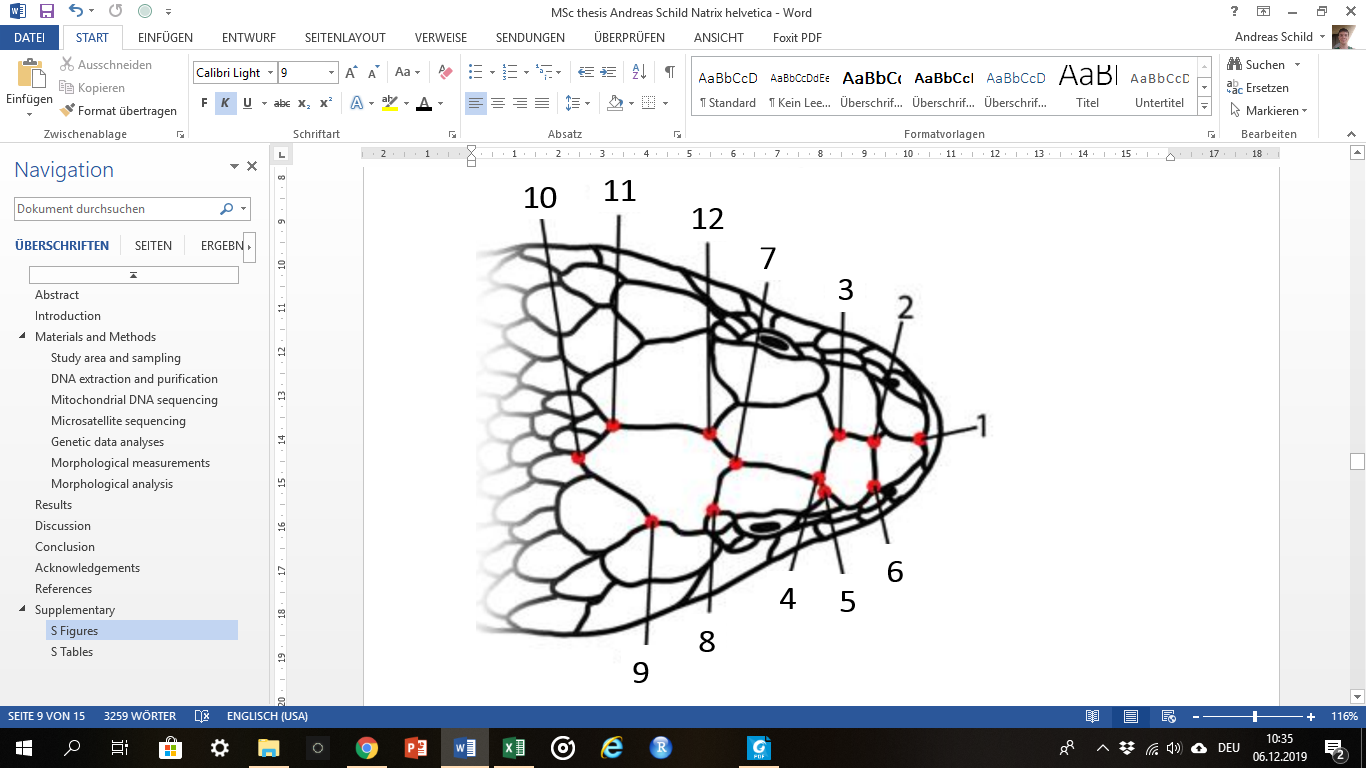

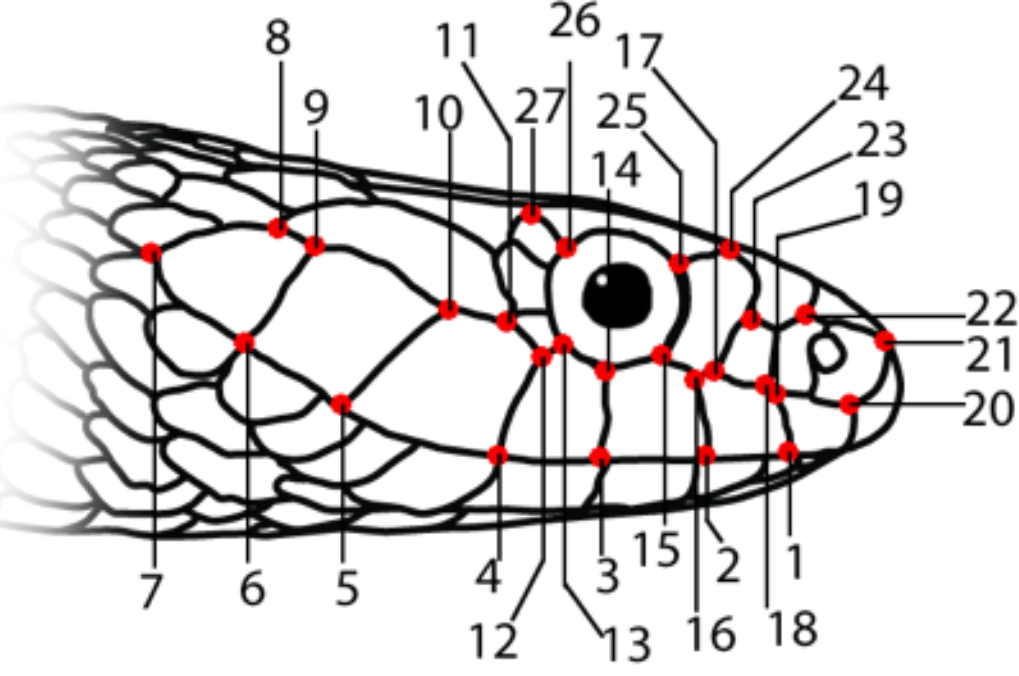


**Figure S1.** Landmark positions. All landmarks were placed at junctions of scales except for 7 and 8 on the lateral side of the head. Landmark 7 is the most posterior tip of the last supralabial scale and landmark 8 is the most posterior point of contact between the temporal scale and a supralabial scale.

| 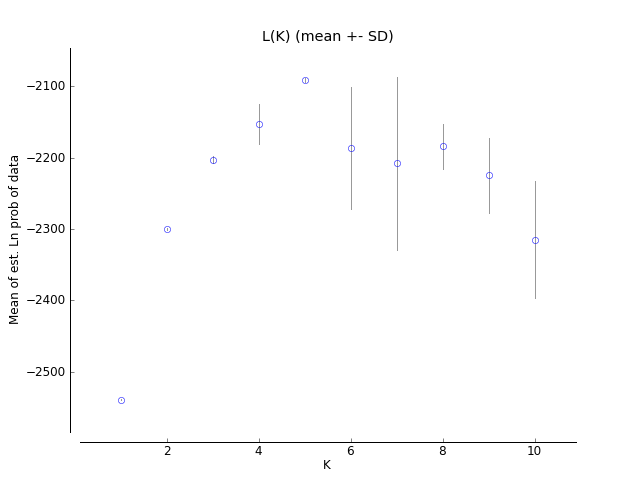 | 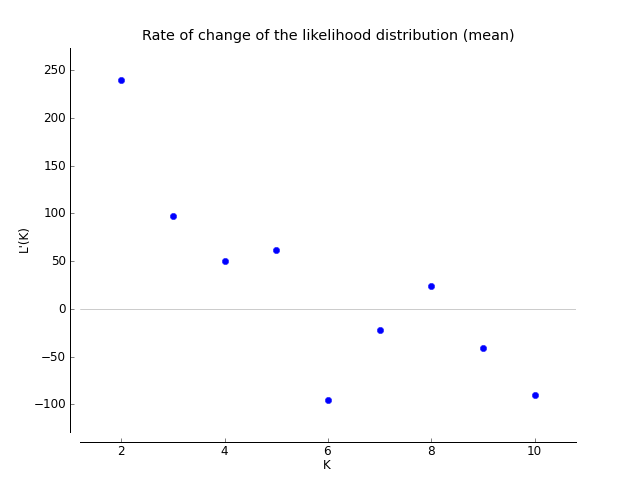 |
| --- | --- |
| 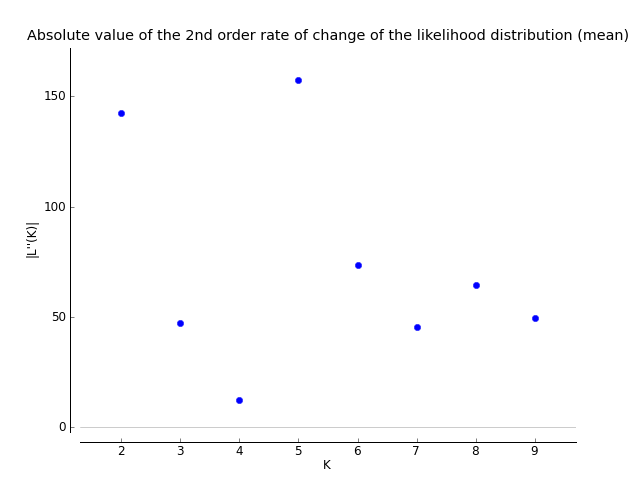 | 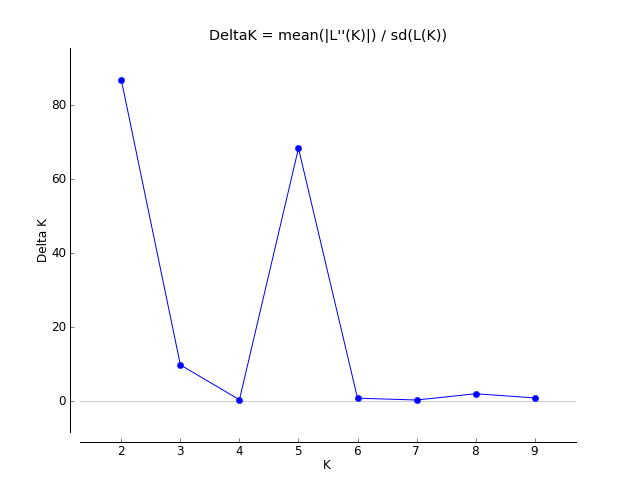 |

**Figure S2.** structure harvester results indicating the optimal number of microsatellite clusters (*K*). According to Pritchard and Wen (2002), the optimal value for *K* has the highest likelihood L(*K*) (top left), which is here *K*=5. The Δ*K* method (Evanno et al. 2005) returns the optimal value with the highest Δ*K* value (bottom right) and identifies the uppermost hierarchical level as *K*=2.


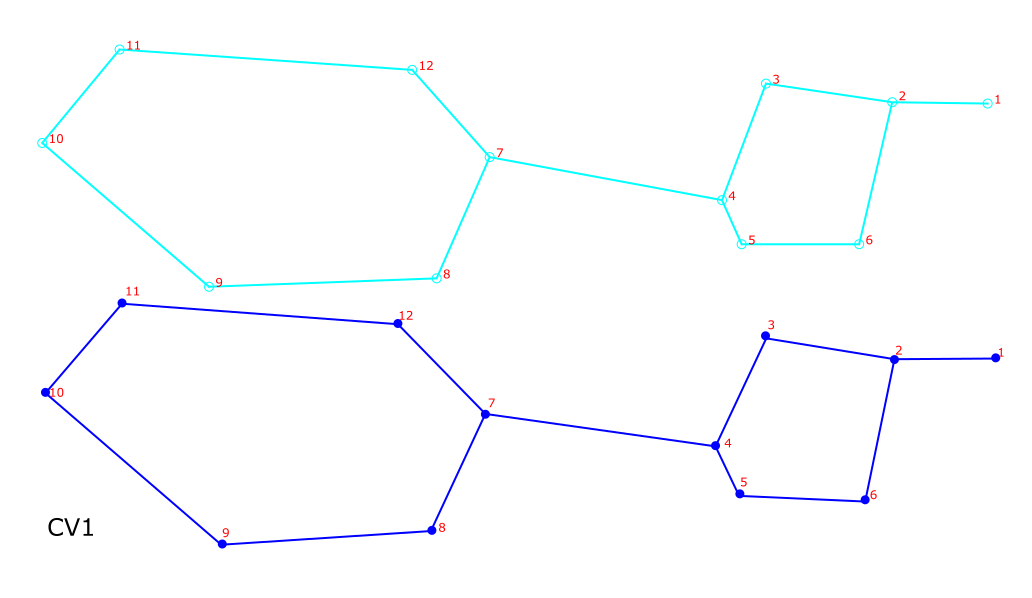


**Figure S3.** Wireframe graph showing shape changes along the canonical variate 1 (CV1) for dorsal landmarks. Light blue shows the starting shape (mean landmark coordinates) and dark blue the target shape (shape shift along CV1). The lower the scores of a specimen for CV1 are, the more does its shape resemble the light blue shape; the higher, the more does its shape resemble the dark blue shape. Accordingly, dorsal landmarks of microsatellite clusters C-VS and C-TI (*Natrix helvetica sicula*) more closely resemble the light blue configuration, while E (*N. h. helvetica*) and *N. natrix* are more similar to the dark blue one (see Fig. 2a for CV scores). Shape changes from light to dark blue are increased threefold.


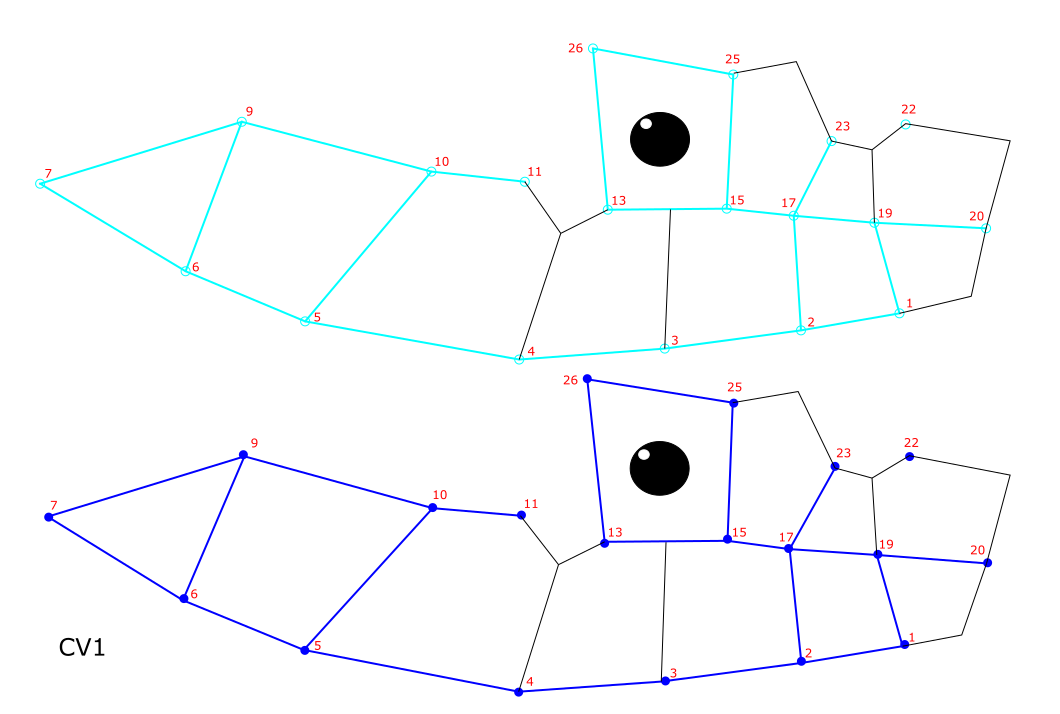


**Figure S4.** Wireframe graph showing the shape changes along the canonical variate 1 (CV1) for lateral landmarks. Light blue shows the starting shape (mean landmark coordinates) and dark blue the target shape (shape shift along CV1). The lower the scores of a specimen for CV1 are, the more does its shape resemble the light blue shape; the higher, the more does its shape resemble the dark blue shape. Accordingly, lateral landmarks of microsatellite clusters resemble the light blue configuration most to least as follows: C-VS > C-TI > E > *N. natrix* (see Fig. 2b for CV scores). Shape changes from light to dark blue are increased threefold.

**Table S1.** Studied grass snake samples. Admixed samples *Natrix helvetica* x *N. natrix* with mito-nuclear discordance are highlighted in red; admixed samples between microsatellite clusters of *N. helvetica* or with mito-nuclear discordance within *N. helvetica*, in blue. Shown are mitochondrial identity (lineage) and proportion of ancestry with microsatellite clusters E-GE, E-ML, C-VS, C-TI (*N. helvetica*) and *N. natrix*, returned by structure; C: Canton. Sample origin: ^1^ A. Schild field sample, ^2^ G. Meier field sample, ^3^ E. Gallice field sample, ^4^ T. Gil field sample, ^5^ S. Dubey field sample, ^6^ Naturhistorisches Museum Bern, ^7^ Museo cantonale di storia naturale Lugano, ^8^ Naturmuseum Sitten, ^9^ M. Chèvre (2015), ^10^ S. Ursenbacher private collection.

| **ID** | **Sex** | **Lineage** | **E-GE** | **E-ML** | **C-VS** | **C-TI** | ***natrix*** | **Canton/ country** | **Community** |
| --- | --- | --- | --- | --- | --- | --- | --- | --- | --- |
| AS001^1^ | M | E | 0.076 | 0.885 | 0.012 | 0.018 | 0.010 | Vaud | Cudrefin |
| AS002^1^ | F | C | 0.007 | 0.027 | 0.801 | 0.116 | 0.050 | Vaud | Noville |
| AS003^1^ | M | C | 0.385 | 0.014 | 0.516 | 0.057 | 0.028 | Vaud | Aigle |
| AS004^1^ | J | C | 0.616 | 0.033 | 0.065 | 0.275 | 0.012 | Vaud | Aigle |
| AS005^1^ | M | E | 0.006 | 0.939 | 0.011 | 0.007 | 0.037 | Vaud | Cudrefin |
| AS006^1^ | J | C | 0.005 | 0.006 | 0.979 | 0.006 | 0.004 | Vaud | Lavey-Morcles |
| AS007^1^ | F | C | 0.003 | 0.005 | 0.984 | 0.004 | 0.004 | Valais | Dorénaz |
| AS008^1^ | F | C | 0.007 | 0.008 | 0.972 | 0.008 | 0.005 | Valais | Martigny |
| AS009^1^ | F | C | 0.006 | 0.006 | 0.976 | 0.006 | 0.006 | Valais | Martigny |
| AS010^1^ | M | C | 0.003 | 0.005 | 0.985 | 0.003 | 0.003 | Valais | Chamoson |
| AS011^1^ | J | C | 0.004 | 0.005 | 0.983 | 0.005 | 0.004 | Valais | Sitten |
| AS012^1^ | M | C | 0.003 | 0.004 | 0.986 | 0.003 | 0.003 | Valais | Sitten |
| AS013^1^ | M | C | 0.004 | 0.006 | 0.979 | 0.008 | 0.004 | Valais | Salgesch |
| AS014^1^ | J | C | 0.003 | 0.003 | 0.988 | 0.003 | 0.003 | Valais | Siders |
| AS015^1^ | M | C | 0.004 | 0.048 | 0.930 | 0.007 | 0.011 | Valais | Ayent |
| AS016^1^ | J | C | 0.004 | 0.026 | 0.944 | 0.009 | 0.016 | Valais | Ayent |
| AS017^1^ | J | C | 0.299 | 0.020 | 0.635 | 0.04 | 0.005 | Vaud | Yvorne |
| AS018^1^ | J | C | 0.035 | 0.218 | 0.704 | 0.024 | 0.019 | Vaud | Yvorne |
| AS019^1^ | F | C | 0.011 | 0.009 | 0.954 | 0.013 | 0.014 | Vaud | Noville |
| AS020^1^ | F | C | 0.011 | 0.019 | 0.948 | 0.017 | 0.005 | Vaud | Noville |
| AS021^1^ | J | C | 0.306 | 0.221 | 0.446 | 0.022 | 0.006 | Vaud | Vevey |
| AS022^1^ | J | E | 0.172 | 0.247 | 0.555 | 0.011 | 0.015 | Vaud | Grandvaux |
| AS023^1^ | M | E | 0.319 | 0.103 | 0.552 | 0.01 | 0.017 | Vaud | Grandvaux |
| AS024^1^ | F | C | 0.013 | 0.014 | 0.948 | 0.016 | 0.010 | Vaud | Château-d'Oex |
| AS025^1^ | J | E | 0.013 | 0.858 | 0.117 | 0.007 | 0.006 | Vaud | Belmont-sur-Lausanne |
| AS026^1^ | F | C | 0.052 | 0.007 | 0.875 | 0.042 | 0.024 | Vaud | Lavey-Morcles |
| AS027^1^ | J | C | 0.192 | 0.016 | 0.626 | 0.14 | 0.025 | Vaud | Noville |
| AS028^1^ | J | C | 0.006 | 0.006 | 0.977 | 0.007 | 0.004 | Vaud | Noville |
| **ID** | **Sex** | **Lineage** | **E-GE** | **E-ML** | **C-VS** | **C-TI** | ***natrix*** | **Canton/ country** | **Community** |
| AS029^1^ | J | 7 | 0.004 | 0.009 | 0.004 | 0.005 | 0.978 | Vaud | Lausanne |
| AS030^1^ | J | C | 0.012 | 0.139 | 0.008 | 0.009 | 0.831 | Vaud | Le Mont-sur-Lausanne |
| AS031^1^ | J | E | 0.008 | 0.886 | 0.090 | 0.006 | 0.010 | Vaud | La Sarraz |
| AS032^1^ | J | C | 0.007 | 0.009 | 0.004 | 0.006 | 0.974 | Vaud | Lausanne |
| AS033^1^ | J | E | 0.029 | 0.875 | 0.028 | 0.059 | 0.009 | Vaud | Arnex-sur-Orbe |
| AS034^1^ | J | C | 0.174 | 0.012 | 0.018 | 0.781 | 0.016 | Ticino | Lavertezzo |
| AS035^1^ | n/a | C | 0.021 | 0.004 | 0.005 | 0.838 | 0.132 | Ticino | Blenio |
| AS036^2^ | J | C | 0.017 | 0.007 | 0.008 | 0.957 | 0.011 | Ticino | Isone |
| AS037^2^ | J | C | 0.013 | 0.004 | 0.004 | 0.974 | 0.005 | Ticino | Isone |
| AS038^2^ | J | C | 0.009 | 0.008 | 0.008 | 0.944 | 0.030 | Italy | Mosso (PIE) |
| AS039^2^ | J | C | 0.006 | 0.006 | 0.012 | 0.971 | 0.005 | Ticino | Camignolo |
| AS040^3^ | J | E | 0.573 | 0.228 | 0.005 | 0.185 | 0.009 | Geneva | Chêne-Bougeries |
| AS041^3^ | J | E | 0.980 | 0.008 | 0.004 | 0.004 | 0.004 | Geneva | Thônex |
| AS042^3^ | J | E | 0.979 | 0.008 | 0.004 | 0.005 | 0.004 | Geneva | Chêne-Bougeries |
| AS043^3^ | J | E | 0.955 | 0.022 | 0.006 | 0.010 | 0.007 | Geneva | Chêne-Bougeries |
| AS044^4^ | J | E | n/a | n/a | n/a | n/a | n/a | Geneva | Chêne-Bougeries |
| AS045^4^ | J | E | 0.959 | 0.018 | 0.006 | 0.011 | 0.006 | Geneva | Chêne-Bougeries |
| AS046^4^ | J | E | 0.581 | 0.392 | 0.007 | 0.014 | 0.006 | Geneva | Chêne-Bougeries |
| AS047^5^ | F | E | 0.940 | 0.015 | 0.003 | 0.020 | 0.022 | Vaud | Trélex |
| AS048^5^ | M | C | 0.261 | 0.581 | 0.133 | 0.017 | 0.008 | Vaud | Allaman |
| AS049^2^ | F | C | 0.192 | 0.030 | 0.022 | 0.690 | 0.065 | Ticino | Airolo |
| NMBE 1016027^6^ | F | E | 0.010 | 0.975 | 0.005 | 0.005 | 0.006 | Bern | Mühleberg |
| NMBE 1021790^6^ | M | C | 0.011 | 0.951 | 0.007 | 0.011 | 0.019 | Bern | Langnau im Emmental |
| NMBE 1048883^6^ | J | E | n/a | n/a | n/a | n/a | n/a | Bern | Aarberg |
| NMBE 1052697^6^ | n/a | E | 0.382 | 0.483 | 0.018 | 0.046 | 0.071 | Bern | Ringgenberg |
| NMBE 1055597^6^ | n/a | E | 0.008 | 0.970 | 0.007 | 0.006 | 0.009 | Bern | Diemtigen |
| NMBE 1055598^6^ | F | E | 0.011 | 0.962 | 0.011 | 0.009 | 0.007 | Bern | Allmendingen |
| NMBE 1055599^6^ | J | E | 0.015 | 0.954 | 0.014 | 0.012 | 0.006 | Bern | Spiez |
| NMBE 1055681^6^ | M | E | 0.021 | 0.941 | 0.011 | 0.007 | 0.020 | Bern | Walperswil |
| NMBE 1055998^6^ | J | E | 0.304 | 0.488 | 0.180 | 0.016 | 0.013 | Bern | Wohlen bei Bern |
| V 1095^7^ | F | C | 0.044 | 0.007 | 0.007 | 0.936 | 0.005 | Italy | Cameri (NO) |
| **ID** | **Sex** | **Lineage** | **E-GE** | **E-ML** | **C-VS** | **C-TI** | ***natrix*** | **Canton/ country** | **Community** |
| V 1121^7^ | J | C | 0.008 | 0.006 | 0.006 | 0.975 | 0.005 | Italy | Cameri (NO) |
| VT 1327^7^ | J | C | n/a | n/a | n/a | n/a | n/a | Ticino | Lugano |
| VT 1332^7^ | F | n/a | 0.008 | 0.029 | 0.007 | 0.923 | 0.034 | Ticino | Agno |
| VT 1326^7^ | M | n/a | n/a | n/a | n/a | n/a | n/a | Ticino | n/a |
| VT 2917^7^ | F | n/a | n/a | n/a | n/a | n/a | n/a | Ticino | Bedigliora |
| VT 5192^7^ | F | n/a | n/a | n/a | n/a | n/a | n/a | Ticino | Alto Malcantone |
| VT 1324^7^ | F | n/a | n/a | n/a | n/a | n/a | n/a | Ticino | n/a |
| VT 1317/2^7^ | J | n/a | n/a | n/a | n/a | n/a | n/a | Ticino | n/a |
| VT 2341^7^ | J | C | 0.028 | 0.012 | 0.023 | 0.926 | 0.010 | Ticino | Rancate |
| VT 1325/1^7^ | M | n/a | n/a | n/a | n/a | n/a | n/a | Ticino | n/a |
| VT 1325/2^7^ | M | n/a | n/a | n/a | n/a | n/a | n/a | Ticino | n/a |
| VT 2421^7^ | F | n/a | n/a | n/a | n/a | n/a | n/a | Ticino | Genestrerio |
| VT 1331^7^ | F | C | 0.061 | 0.015 | 0.056 | 0.841 | 0.026 | Ticino | Lumino |
| CCTC 2000539^8^ | F | C | 0.005 | 0.014 | 0.973 | 0.004 | 0.004 | Valais | Saillon |
| CCTC 2000540^8^ | J | C | 0.009 | 0.018 | 0.005 | 0.961 | 0.007 | Italy | Verrès (AO) |
| CCTC 2010517^8^ | F | C | 0.030 | 0.023 | 0.930 | 0.011 | 0.006 | Valais | Vernayaz |
| CCTC 9631^8^ | M | C | 0.007 | 0.008 | 0.974 | 0.006 | 0.005 | Valais | Martigny |
| BAL02^9^ | M | E | n/a | n/a | n/a | n/a | n/a | St. Gallen | Widnau |
| BAL04^9^ | M | E | n/a | n/a | n/a | n/a | n/a | St. Gallen | Widnau |
| DIE03^9^ | F | E | n/a | n/a | n/a | n/a | n/a | St. Gallen | Diepoldsau |
| DUB01^9^ | F | E | n/a | n/a | n/a | n/a | n/a | Zürich | Volketswil |
| DUB02^9^ | M | E | n/a | n/a | n/a | n/a | n/a | Zürich | Volketswil |
| DUB03^9^ | F | E | n/a | n/a | n/a | n/a | n/a | Zürich | Volketswil |
| DUB04^9^ | F | E | n/a | n/a | n/a | n/a | n/a | Zürich | Volketswil |
| GIP03^9^ | F | E | n/a | n/a | n/a | n/a | n/a | Aargau | Leuggern |
| GIP04^9^ | F | E | n/a | n/a | n/a | n/a | n/a | Aargau | Leuggern |
| GIP05^9^ | F | E | n/a | n/a | n/a | n/a | n/a | Aargau | Leuggern |
| KGN01^9^ | F | E | n/a | n/a | n/a | n/a | n/a | Aargau | Böttstein |
| KLI01^9^ | F | E | n/a | n/a | n/a | n/a | n/a | Aargau | Klingnau |
| ROH01^9^ | F | E | n/a | n/a | n/a | n/a | n/a | Aargau | Aarau |
| ROH02^9^ | M | E | n/a | n/a | n/a | n/a | n/a | Aargau | Aarau |
| ROH04^9^ | F | E | n/a | n/a | n/a | n/a | n/a | Aargau | Aarau |
| STA01^9^ | F | E | n/a | n/a | n/a | n/a | n/a | Zürich | Hombrechtikon |
| STA02^9^ | M | E | n/a | n/a | n/a | n/a | n/a | Zürich | Hombrechtikon |
| ZHC01^9^ | M | E | n/a | n/a | n/a | n/a | n/a | Zürich | Zürich |
| **ID** | **Sex** | **Lineage** | **E-GE** | **E-ML** | **C-VS** | **C-TI** | ***natrix*** | **Canton/ country** | **Community** |
| ZHC02^9^ | M | E | n/a | n/a | n/a | n/a | n/a | Zürich | Zürich |
| ZHN01^9^ | M | E | n/a | n/a | n/a | n/a | n/a | Zürich | Zürich |
| ZHN02^9^ | M | E | n/a | n/a | n/a | n/a | n/a | Zürich | Zürich |
| AAD01^9^ | F | 3 | n/a | n/a | n/a | n/a | n/a | Thurgau | Aadorf |
| BAD01^9^ | F | 3 | n/a | n/a | n/a | n/a | n/a | Aargau | Bad Zurzach |
| BAD02^9^ | M | 3 | n/a | n/a | n/a | n/a | n/a | Aargau | Bad Zurzach |
| BAD06^9^ | M | 3 | n/a | n/a | n/a | n/a | n/a | Aargau | Bad Zurzach |
| FCH02^9^ | F | 3 | n/a | n/a | n/a | n/a | n/a | Zürich | Flaach |
| FCH04^9^ | M | 3 | n/a | n/a | n/a | n/a | n/a | Zürich | Flaach |
| KLO02^9^ | M | 3 | n/a | n/a | n/a | n/a | n/a | Zürich | Rümlang |
| KLO07^9^ | F | 3 | n/a | n/a | n/a | n/a | n/a | Zürich | Rümlang |
| MAR01^9^ | M | 3 | n/a | n/a | n/a | n/a | n/a | Zürich | Rheinau |
| MAR02^9^ | F | 3 | n/a | n/a | n/a | n/a | n/a | Zürich | Rheinau |
| NEB02^9^ | F | 3 | n/a | n/a | n/a | n/a | n/a | St. Gallen | St. Margrethen |
| NEB04^9^ | M | 3 | n/a | n/a | n/a | n/a | n/a | St. Gallen | St. Margrethen |
| NEE03^9^ | M | 3 | n/a | n/a | n/a | n/a | n/a | Zürich | Höri |
| NWN01^9^ | M | 3 | n/a | n/a | n/a | n/a | n/a | Thurgau | Kemmental |
| NWN02^9^ | F | 3 | n/a | n/a | n/a | n/a | n/a | Thurgau | Kemmental |
| OBE04^9^ | M | 3 | n/a | n/a | n/a | n/a | n/a | St. Gallen | Oberuzwil |
| RIM00^9^ | F | 3 | n/a | n/a | n/a | n/a | n/a | Aargau | Rietheim |
| SHF01^9^ | F | 3 | n/a | n/a | n/a | n/a | n/a | Schaffhausen | Schaffhausen |
| SHO02^9^ | M | 3 | n/a | n/a | n/a | n/a | n/a | Schaffhausen | Hallau |
| SHO03^9^ | F | 3 | n/a | n/a | n/a | n/a | n/a | Schaffhausen | Hallau |
| NN1017^10^ | n/a | C | n/a | n/a | n/a | n/a | n/a | Grisons | Val Bregaglia, Bondo |
| NN1041^10^ | n/a | 7 | 0.005 | 0.007 | 0.006 | 0.007 | 0.975 | Vaud | Lausanne |
| NN1042^10^ | n/a | 7 | 0.010 | 0.023 | 0.011 | 0.006 | 0.950 | Vaud | Lausanne |
| NN1049^10^ | n/a | C | n/a | n/a | n/a | n/a | n/a | Vaud | Lausanne |
| NN1053^10^ | n/a | C | 0.046 | 0.838 | 0.007 | 0.008 | 0.100 | Vaud | Epalinges |
| NN1054^10^ | n/a | C | 0.038 | 0.109 | 0.770 | 0.019 | 0.064 | Vaud | Ollon |
| NN1055^10^ | n/a | n/a | 0.013 | 0.027 | 0.833 | 0.016 | 0.112 | Vaud | Villeneuve |
| NN1056^10^ | n/a | E | 0.788 | 0.021 | 0.006 | 0.155 | 0.030 | Vaud | Grandvaux |
| NN1057^10^ | n/a | E | n/a | n/a | n/a | n/a | n/a | Vaud | Chamblon |
| NN1058^10^ | n/a | C | 0.005 | 0.007 | 0.976 | 0.007 | 0.004 | Vaud | Ollon |
| NN1060^10^ | n/a | C | 0.005 | 0.008 | 0.977 | 0.005 | 0.005 | Vaud | Ollon |
| NN1061^10^ | n/a | C | n/a | n/a | n/a | n/a | n/a | Valais | Sierre |
| NNB01^10^ | n/a | E | n/a | n/a | n/a | n/a | n/a | Bern | Ins |
| **ID** | **Sex** | **Lineage** | **E-GE** | **E-ML** | **C-VS** | **C-TI** | ***natrix*** | **Canton/ country** | **Community** |
| NNB02^10^ | n/a | E | n/a | n/a | n/a | n/a | n/a | Bern | Ins |
| NNB03^10^ | n/a | E | n/a | n/a | n/a | n/a | n/a | Bern | Muri |
| NNB04^10^ | n/a | E | n/a | n/a | n/a | n/a | n/a | Bern | Gadmental |
| NNB06^10^ | n/a | C | n/a | n/a | n/a | n/a | n/a | Schwyz | Muotathal |
| NNB07^10^ | n/a | E | n/a | n/a | n/a | n/a | n/a | Vaud | Vinzel |

n/a = not available

**Table S2.** Morphological variables measured. Illustrations from Chèvre (2015).

| **Variable** | **Code** | **Notes** | **Unit** | **Illustration** |
| --- | --- | --- | --- | --- |
| Snout-vent length | SVL | From tip of snout to cloaca. Snake was hold next to a ruler to measure (in the field) | cm |  |
| Tail length | TL | From cloaca to end of tail; only for intact tail. Snake was hold next to a ruler to measure (in the field) | cm |  |
| Body weight | BW | Using a kitchen scales (precision: ± 0.5 g). Not for museum specimens. Done in the field | g |  |
| Head length | HL | From tip of snout to inflexion point at the end of the mandible. Mean of lengths to left and right mandible | cm | 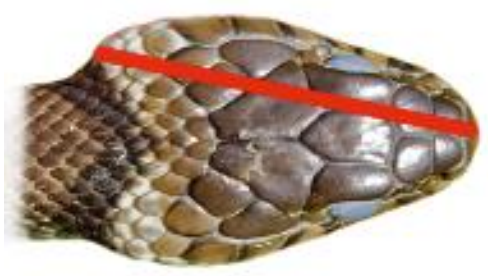 |
| Head width | HW | Distance between posterior tips of last supralabial scale | cm | 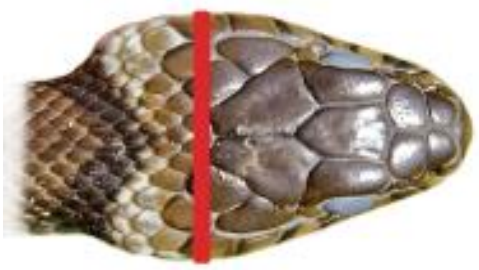 |
| Number of ventral scales | VS | Ventral scales are wider than long, from head to cloaca. Counted in the field |  | 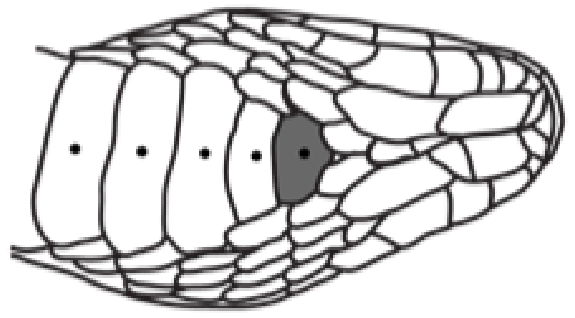 |
| Relative position of the reduction from 19 to 17 dorsal scale rows | RelRedPos | % of the number of ventral scales to the position of reduction (starting from the head) from the total number of ventral scales. Counted in the field |  | 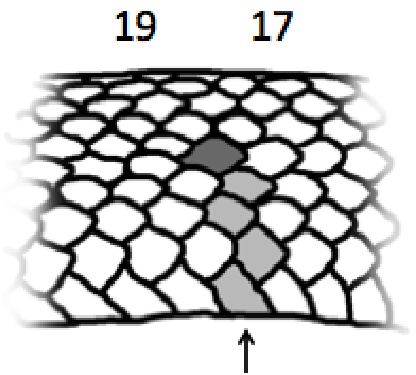 |
| Number of paired subcaudal scales | SCS | From cloaca to tip of tail. Counted in the field |  |  |
| **Variable** | **Code** | **Notes** | **Unit** | **Illustration** |
| Number of contacts between temporal and lower post-ocular scale | TPOS | Sum of both sides -> 0, 1 or 2 |  | 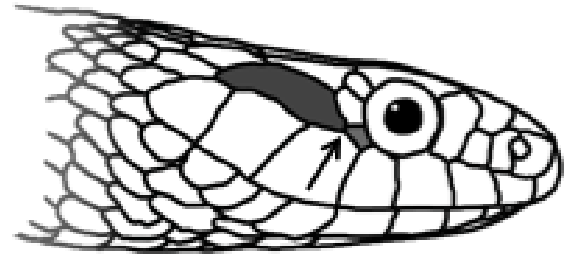 |
| Number of post-temporal scales | PTS | Sum of both sides |  | 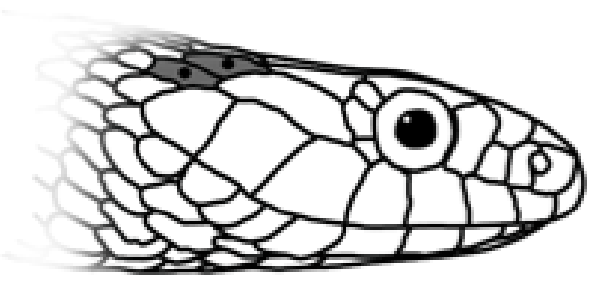 |
| Number of gular scales | GS |  |  | 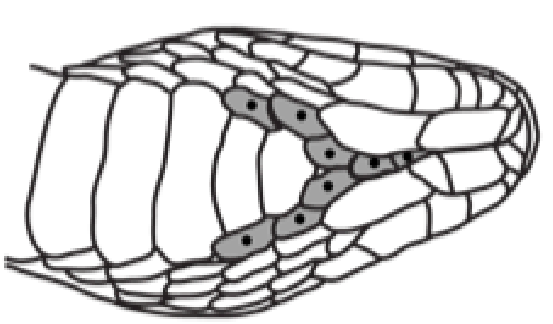 |
| Lateral blotches size | LBS | Number of scales filled by the blotch |  | 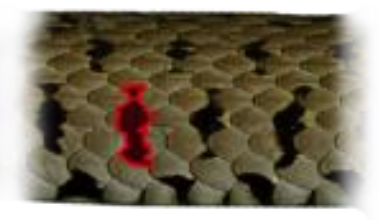 |
| Lateral blotches length | LBL | Number of scale widths  (NOT number of scale rows) |  | 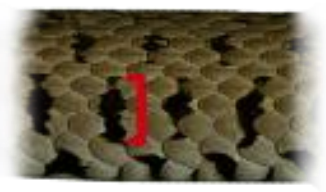 |
| Lateral blotches width | LBW | Number of scale lengths  (NOT number of scale rows) |  | 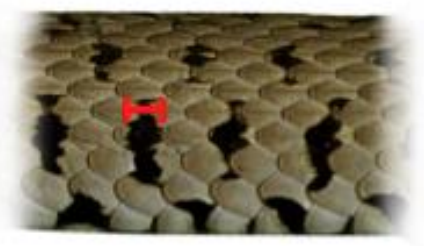 |
| Nuchal marking size | NMS | Number of scales filled by the marking |  | 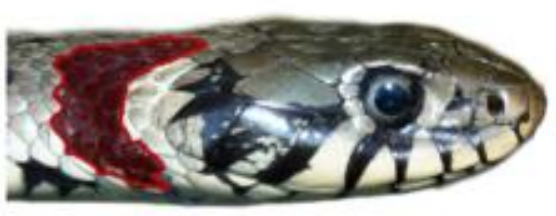 |
| Nuchal marking width | NMW | Number of scale lengths at widest point  (NOT number of scale rows) |  | 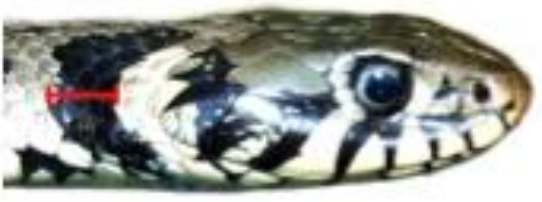 |
| Upper curvature of nuchal marking | NMUC | Number of scales exceeding midpoint of marking (dorsal) |  | 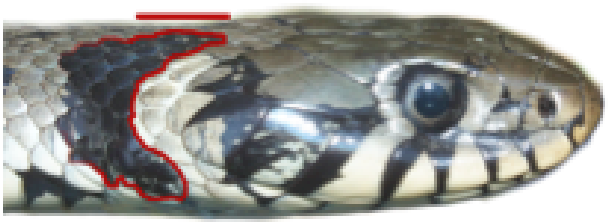 |
| Lower curvature of nuchal marking | NMLC | Number of scales exceeding midpoint of marking (ventral) |  | 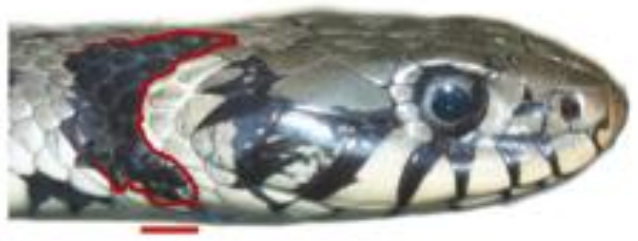 |
| Distance between nuchal marking and parietal scales | NMPS | Number of scale lengths between midpoint of nuchal marking and posterior tips of parietal scales |  | 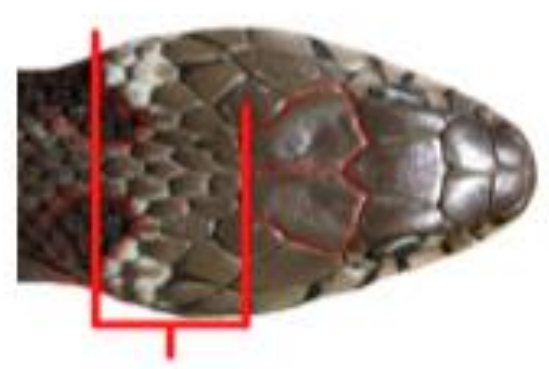 |

**Table S3.** Pairwise *F*_ST_ values between microsatellite clusters of *Natrix helvetica* (E-GE, E-ML, C-VS and C-TI) and *N. natrix*. Values were calculated with FSTAT ver. 2.9.3 (Goudet 1995) including only individuals with a microsatellite assignment to one cluster ≥ 80%.

| **E-GE** | **E-ML** | **C-VS** | **C-TI** | ***natrix*** |  |
| --- | --- | --- | --- | --- | --- |
| 0.000 |  |  |  |  | E-GE |
| 0.150 | 0.000 |  |  |  | E-ML |
| 0.282 | 0.180 | 0.000 |  |  | C-VS |
| 0.197 | 0.196 | 0.197 | 0.000 |  | C-TI |
| 0.199 | 0.139 | 0.188 | 0.194 | 0.000 | *natrix* |

**Table S4.** Morphological assignment of specimens (horizontal rows) to microsatellite clusters of *Natrix natrix* and *N. helvetica* (E, C-VS, C-TI, vertical columns) based on all morphological variables using a Linear Discriminant Analysis (LDA). The percentages are correctly classified individuals according to their genetic identity. Left: with *N. natrix*, right: without *N. natrix*.

|  | ***natrix*** | **E** | **C-VS** | **C-TI** | **%** |  |  | **E** | **C-VS** | **C-TI** | **%** |
| --- | --- | --- | --- | --- | --- | --- | --- | --- | --- | --- | --- |
| *natrix* | 13 | 2 | 0 | 0 | 86.7 |  | E | 13 | 5 | 6 | 54.2 |
| E | 2 | 12 | 6 | 4 | 50.0 |  | C-VS | 3 | 8 | 2 | 61.5 |
| C-VS | 0 | 3 | 9 | 1 | 69.2 |  | C-TI | 4 | 1 | 3 | 37.5 |
| C-TI | 0 | 3 | 2 | 3 | 37.5 |  |  |  |  |  |  |

**Table S5.** Mean and standard deviation (SD) of different morphological traits for Swiss barred grass snakes (*Natrix helvetica*).

| **Variable** | ***N. h. helvetica* (mean ± SD)** | ***N. h. sicula***  **(mean ± SD)** |
| --- | --- | --- |
| Snout-vent length [cm] | 60.81 ± 16.30 | 64.00 ± 14.20 |
| Tail length [cm] | 14.03 ± 2.79 | 15.70 ± 2.75 |
| Body weight [g] | 139.40 ± 118.10 | 178.00 ± 128.54 |
| Head length [cm] | 2.80 ± 0.71 | 2.92 ± 0.72 |
| Head width [cm] | 1.57 ± 0.48 | 1.55 ± 0.43 |
| Number of ventral scales | 172.66 ± 4.96 | 173.83 ± 3.69 |
| Relative position of the reduction from 19 to 17 dorsal scale rows | 0.53 ± 0.04 | 0.52 ± 0.08 |
| Number of paired subcaudal scales | 60.44 ± 6.49 | 64.68 ± 8.68 |
| Number of contacts between temporal and lower post-ocular scale | 1.52 ± 0.80 | 1.58 ± 0.72 |
| Number of post-temporal scales | 4.66 ± 1.04 | 4.75 ± 0.90 |
| Number of gular scales | 8.19 ± 1.42 | 7.83 ± 0.82 |
| Lateral blotches size | 1.37 ± 0.66 | 1.57 ± 0.71 |
| Lateral blotches length | 2.33 ± 1.15 | 2.58 ± 0.81 |
| Lateral blotches width | 0.66 ± 0.16 | 0.62 ± 0.24 |
| Nuchal marking size | 26.35 ± 4.26 | 24.98 ± 4.45 |
| Nuchal marking width | 4.11 ± 0.49 | 3.87 ± 0.50 |
| Upper curvature of nuchal marking | 0.94 ± 0.41 | 0.73 ± 0.34 |
| Lower curvature of nuchal marking | 1.27 ± 0.34 | 1.34 ± 0.38 |
| Distance between nuchal marking and parietal scales | 3.44 ± 0.42 | 3.54 ± 0.52 |
